# Differential diagnosis of pneumoconiosis mass shadows and peripheral lung cancer using CT radiomics and the AdaBoost machine learning model

**DOI:** 10.3389/fmed.2025.1675840

**Published:** 2025-12-03

**Authors:** Xiaobing Li, Wei Wang, Xuemei Li, Qianqian Liu, Yongsheng Liu, Li Wang, Qian Li, Li Zhang, Wutao Xie

**Affiliations:** 1 Science and Technology Industry Development Center, Chongqing Medical and Pharmaceutical College, Chongqing, China; 2 Laboratory of Toxicology, The First Affiliated Hospital of Chongqing Medical and Pharmaceutical College, Chongqing, China; 3 Chongqing Key Laboratory of Prevention and Treatment for Occupational Diseases and Poisoning, The First Affiliated Hospital of Chongqing Medical and Pharmaceutical College, Chongqing, China; 4 Department of Occupational Disease and Poisoning Medicine, The First Affiliated Hospital of Chongqing Medical and Pharmaceutical College, Chongqing, China; 5 College of Public Health, Chongqing Medical University, Chongqing, China; 6 Department of Radiology, The First Affiliated Hospital of Chongqing Medical and Pharmaceutical College, Chongqing, China; 7 NHC Key Laboratory of Diagnosis and Treatment on Brain Functional Diseases, Department of Neurology, First Affiliated Hospital of Chongqing Medical University, Chongqing, China

**Keywords:** pneumoconiosis, large opacities, CT radiomics, AdaBoost, machine learning, diagnostic model

## Abstract

**Objective:**

To develop a differential diagnostic prediction model for distinguishing large opacities in pneumoconiosis from peripheral lung cancer based on CT radiomics.

**Methods:**

A total of 103 cases of large opacities in pneumoconiosis and 85 cases of peripheral lung cancer were retrospectively collected from routine CT scans at the First Affiliated Hospital of Chongqing Medical and Pharmaceutical College between March 2021 and June 2025. Diagnosis was confirmed by an expert panel, clinical evaluations, and pathological examinations. Patients were randomly assigned to a training set (*n* = 132) and a test set (*n* = 56). Lesions were delineated by at least two pneumoconiosis experts using ITK-SNAP software. Radiomic features were extracted from CT images of lung lesions in the training set, including first-order features, shape features (2D and 3D), texture features (gray-level co-occurrence matrix, gray-level run-length matrix, gray-level size-zone matrix, gray-level dependence matrix), and wavelet transform filters. Feature dimensionality reduction was applied to construct morphological biomarkers. Diagnostic prediction models were built using machine learning algorithms. Model performance was evaluated using the ROC curve and the area under the curve (AUC) in the test set.

**Results:**

A total of 108 features were extracted from 110 large opacity regions and 85 peripheral lung cancer regions of interest (ROIs). Dimensionality reduction identified a subset of eight most significant features. LR, SVM, and AdaBoost algorithms were implemented using Python to build the models. In the training set, the accuracies of the LR, SVM, and AdaBoost models were 79.4, 84.0, and 80.9%, respectively; the sensitivities were 74.1, 74.1, and 81.0%, respectively; the specificities were 83.6, 91.8, and 80.8%, respectively; and the AUC values were 0.837, 0.886, and 0.900, respectively. In the test set, the accuracies of the LR, SVM, and AdaBoost models were 80.7, 82.5, and 86.0%, respectively; the sensitivities were 89.3, 89.3, and 82.1%, respectively; the specificities were 72.4, 75.9, and 89.7%, respectively; and the AUC values were 0.825, 0.855, and 0.900, respectively. The AUC of the AdaBoost ROC curve was significantly superior to those of the LR and SVM models. The AdaBoost model demonstrated the optimal predictive performance in both the training and test sets.

**Conclusion:**

The AdaBoost-based prediction model, developed using CT radiomic features, effectively differentiates large opacities of stage III occupational pneumoconiosis from peripheral lung cancer.

## 1 Introduction

Pneumoconiosis is a progressive lung disease caused by the long-term inhalation of harmful mineral dusts, typically encountered in occupational settings such as mining, construction, and manufacturing (1). The dust particles become lodged in the pulmonary tissue, leading to inflammation, fibrosis, and the eventual development of severe pulmonary dysfunction (2). The disease primarily manifests as diffuse pulmonary fibrosis, which can progress to more severe forms, including progressive massive fibrosis (PMF) in advanced stages (3). PMF is characterized by large opacities in the lungs, often exceeding 10 mm in diameter, as defined by the National Occupational Health Standard of the People’s Republic of China (4). In stage III pneumoconiosis, these large opacities present with a long diameter of at least 20 mm and a short diameter greater than 10 mm. Clinically, patients with advanced pneumoconiosis, particularly those with PMF, experience progressive respiratory symptoms such as dyspnea, chronic cough, and a significant decline in lung function (5). These clinical manifestations are not only debilitating but also share a striking resemblance to those seen in patients with peripheral lung cancer, which complicates the differentiation between the two diseases (6).

Lung cancer, especially in its peripheral form, is one of the most common and lethal malignancies worldwide. Peripheral lung cancer typically originates from the distal bronchioles or alveolar epithelium and often presents as solitary pulmonary nodules or masses located near the pleura (7). In its early stages, the disease may be asymptomatic or manifest with non-specific respiratory symptoms such as cough or chest pain (8). As the tumor progresses, features such as pleural indentation, vascular convergence, spiculation, and local invasion become more apparent. These radiologic characteristics, however, can closely mimic those of PMF, particularly when large opacities are present (9). In addition, patients with pneumoconiosis who are at risk due to occupational exposure may concurrently develop lung cancer, further complicating diagnosis (9). Unlike PMF, which generally follows a chronic, fibrotic course, peripheral lung cancer tends to grow rapidly and metastasize early, leading to poor prognosis if not identified and treated in a timely manner (10).

The overlapping clinical and radiologic features between pneumoconiosis and lung cancer present significant diagnostic challenges. Imaging studies, especially chest X-rays and CT scans, often reveal large opacities that can mimic lung cancer masses in terms of their size, shape, and density (11). As a result, distinguishing between the two conditions solely based on traditional imaging techniques becomes increasingly difficult (10). Therefore, there is an urgent need for accurate diagnostic tools that can help clinicians differentiate between these two conditions effectively (12).

Radiomics, a rapidly evolving field in medical imaging, offers significant promise in addressing this diagnostic dilemma. Radiomics involves the extraction of a wide range of quantitative features from medical images, such as CT scans, to capture the complex spatial and textural characteristics of tissue. These features go beyond traditional imaging analysis and provide detailed insights into the underlying pathological changes in the tissue (13). Lambin et al. first proposed the concept of radiomics in 2012, highlighting its potential to extract clinically relevant information from imaging data that may not be immediately apparent to the human eye (14). By applying advanced machine learning (ML) algorithms to radiomic features, it is possible to develop predictive models that can aid in non-invasive disease diagnosis, prognosis, and treatment planning (15).

In the context of pneumoconiosis and lung cancer, radiomics offers substantial diagnostic value owing to the remarkable similarity in imaging characteristics between progressive massive fibrosis (PMF) and peripheral lung tumors (16). The primary objective of this study was to develop a differential diagnostic prediction model based on radiomic features extracted from CT images of pulmonary lesions. By identifying distinct quantitative patterns that differentiate pneumoconiosis-related large opacities from malignant nodules, we aimed to enhance diagnostic precision and assist clinical decision-making (17).

Leveraging the capacity of machine learning algorithms to analyze high-dimensional data and capture complex, non-linear relationships, our approach provides a robust and scalable framework for improving diagnostic discrimination between these two clinically overlapping conditions (18). This is particularly important given that misdiagnosis can lead to inappropriate management, delayed treatment, or unnecessary invasive procedures (19). Overall, this study addresses a critical unmet need in the field of occupational lung disease and oncologic imaging. By integrating radiomics with advanced computational modeling, our work contributes to the development of a standardized, non-invasive, and reproducible diagnostic tool capable of supporting accurate differentiation between pneumoconiosis and peripheral lung cancer, ultimately improving patient outcomes and guiding more informed clinical practice.

## 2 Materials and methods

### 2.1 General data

Chest CT images of patients with pneumoconiosis-related large opacities and peripheral lung cancer were retrospectively collected from the First Affiliated Hospital of Chongqing Medical and Pharmaceutical College between March 2021 and June 2025. The study cohort was divided into two groups based on clinical and diagnostic criteria: Pneumoconiosis large opacity group: Patients diagnosed with stage III occupational pneumoconiosis presenting with large opacities, as confirmed by an expert panel in accordance with the GBZ70–2015 Diagnostic Criteria for Pneumoconiosis. Lesions were required to have a long-axis diameter ≥ 20 mm and a short-axis diameter > 10 mm. Peripheral lung cancer group: patients with peripheral lung cancer confirmed by histopathological examination, exhibiting mass lesions with a maximum diameter ≥ 30 mm.

**FIGURE 1:**
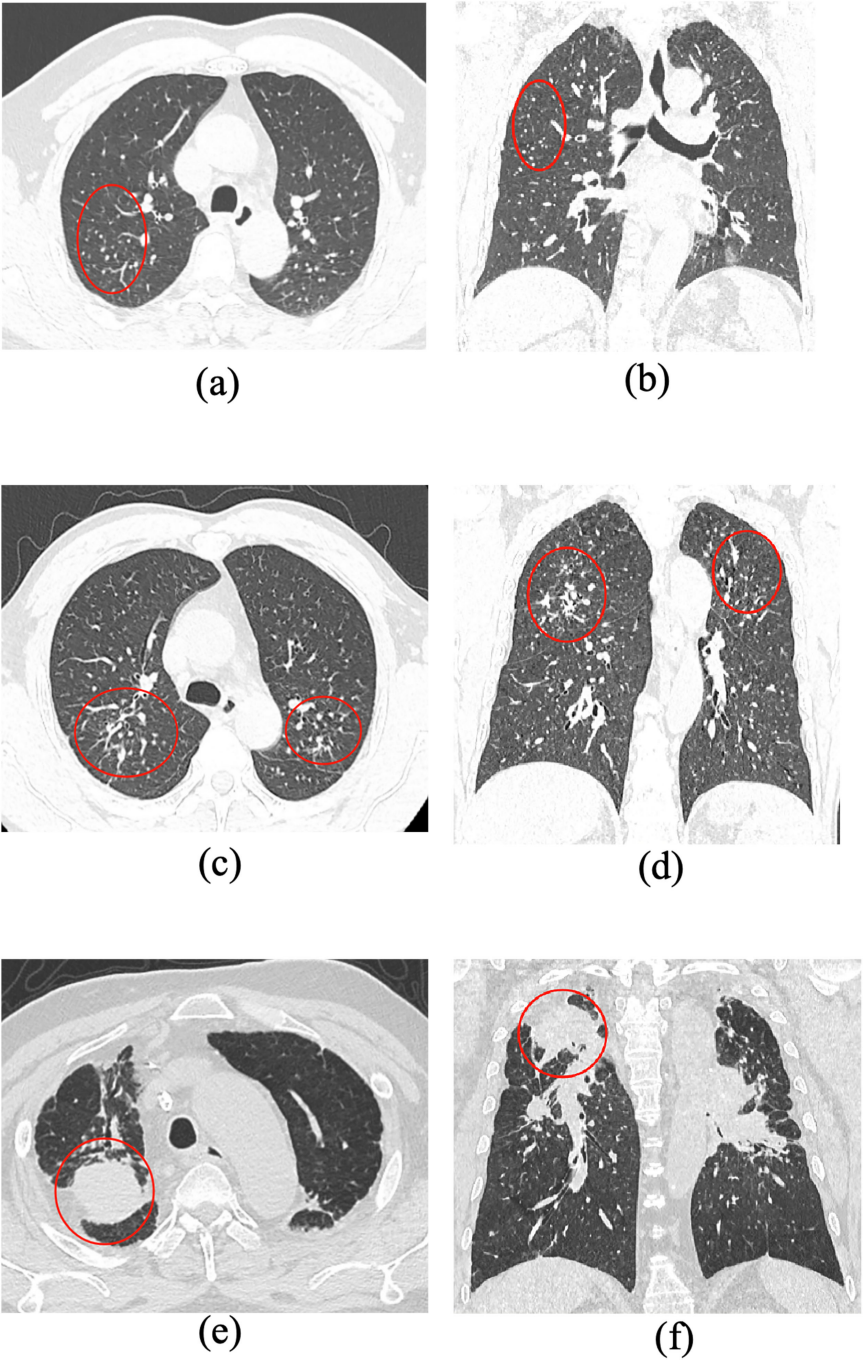
Representative CT imaging features across different stages of pneumoconiosis (axial and coronal views). **(a,b)** Axial and coronal chest CT images from a patient with Stage I pneumoconiosis, revealing multiple small, round, high-density nodules predominantly distributed in the upper lobes of both lungs, especially in the right upper and middle fields. **(c,d)** Correspond to Stage II pneumoconiosis, characterized by an increased number of opacities involving both upper lung zones and the dorsal segment of the lower lobes. **(e,f)** Illustrate stage III pneumoconiosis, showing a homogeneous mass-like opacity in the apicoposterior segment of the right upper lobe, accompanied by scattered calcifications, pleural thickening, adjacent localized emphysema, and nodular interstitial markings. Notably, large opacities (long-axis diameter > 20 mm, short-axis diameter > 10 mm) in advanced pneumoconiosis tend to display asymmetric distribution, suggesting progressive fibrotic remodeling of lung parenchyma.

**FIGURE 2:**
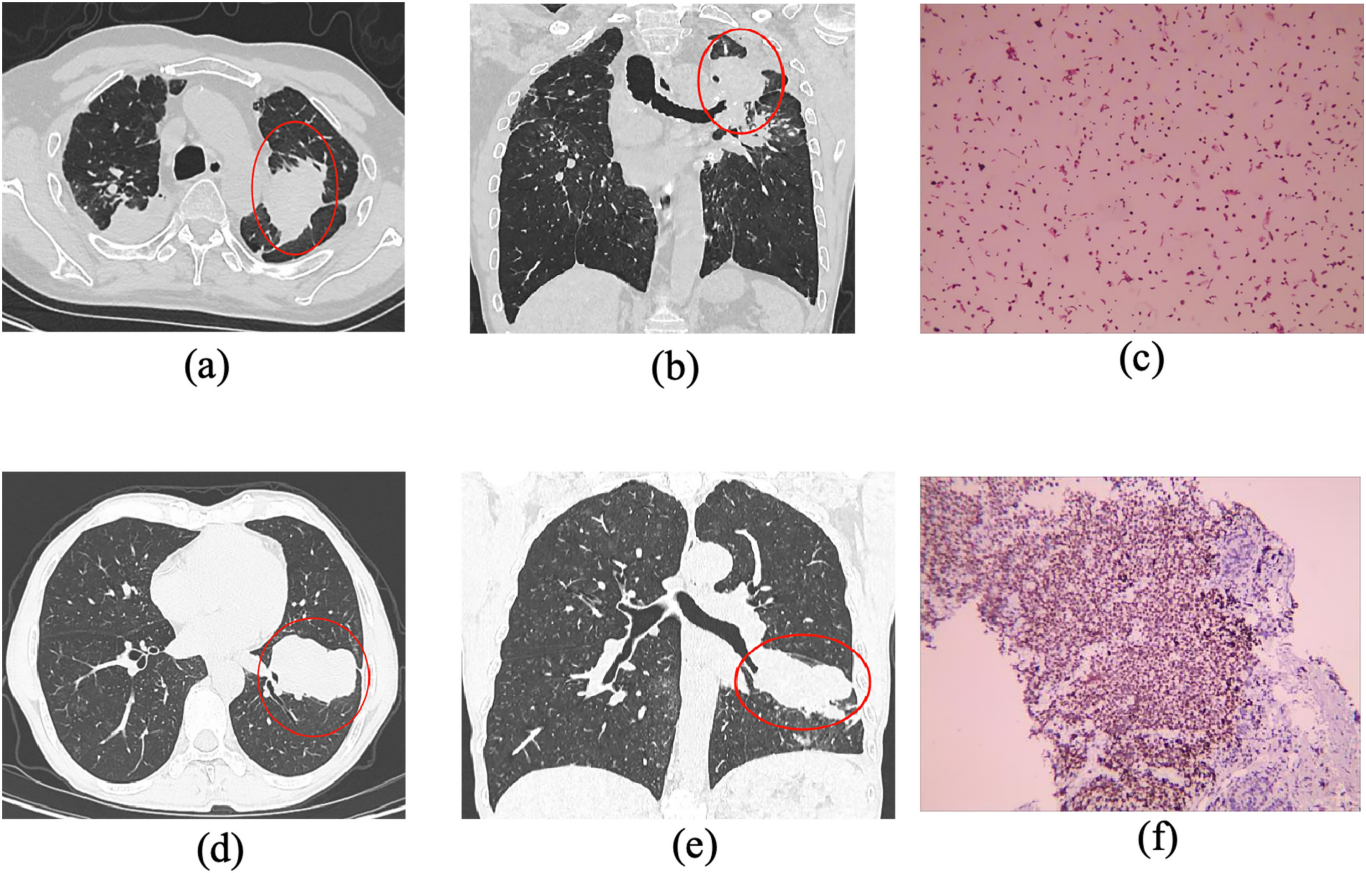
CT and histopathological features of pneumoconiosis-associated and malignant lesions in advanced pneumoconiosis. **(a,b)** Axial and coronal CT images of a Stage III pneumoconiosis patient, demonstrating bilateral apical mass lesions, with a dominant lesion located in the posterior segment of the left upper lobe (long-axis diameter > 20 mm, short-axis diameter > 10 mm). The lesion exhibits irregular margins, peripheral fibrotic strands, pleural thickening, and localized adhesion, along with partial truncation of the left upper bronchus. **(c)** The histopathological examination of the lesion, revealing a heterogeneous composition of epithelial cells and inflammatory infiltrates, predominantly macrophages (45%), lymphocytes (40%), neutrophils (10%), and a minor fraction of other cell types (5%). **(d,e)** Depict a large, multilobulated mass in the posterior segment of the right lower lobe, showing pleural retraction and areas with ill-defined borders. **(f)** Demonstrates immunohistochemical (IHC) staining results, confirming the presence of a malignant neoplasm with focal necrosis. The tumor cells were partially positive for cytokeratin (CK), weakly positive for chromogranin A (CgA) and synaptophysin (Syn), partially positive for INSM1, and sporadically positive for CD56, collectively supporting a diagnosis of neuroendocrine carcinoma.

Inclusion criteria were as follows: (1) For the pneumoconiosis group: confirmed diagnosis of stage III pneumoconiosis with large opacities meeting the size thresholds (long-axis ≥ 20 mm, short-axis > 10 mm) (Figure 1; Supplementary Figure S1). (2) For the lung cancer group: confirmed diagnosis of peripheral lung cancer by both imaging and pathology, with lesions ≥ 30 mm in diameter (Figure 2; Supplementary Figure S2).

Exclusion criteria were defined to ensure image quality and diagnostic specificity: (1) For the pneumoconiosis group: cases with suboptimal CT image quality that failed to meet diagnostic imaging standards, or cases with coexisting pulmonary tuberculosis, metastatic lesions, or other respiratory comorbidities that could confound radiomic analysis. (2) For the lung cancer group: patients with concurrent tuberculosis, pulmonary metastases, or other comorbidities potentially interfering with imaging interpretation.

### 2.2 Methods

#### 2.2.1 CT examination

All subjects underwent CT scans using one of the following devices: GE 128-slice 256-layer Revolution ES Spiral CT (United States), GE Optima CT680, or United Imaging uMI Panorama 860 160-slice CT. Scans were performed from the bilateral lung apices to the lung bases for all patients. The scanning procedure was as follows: ➀ verify patient ID; ➁ prepare the patient; ➂ position the patient supine; ➃ instruct the patient to hold their breath during the scan; ➄ perform the scan; ➅ end the scan.

Scanning parameters: ➀ tube voltage: 120 kV; ➁ rotation time: 0.5 s/rotation; ➂ pitch: 0.991:1; (11) tube current: Auto mA; ➃ scan slice thickness and interval: 5 mm ➄ reconstruction slice thickness: 0.625 mm; ➅ reconstruction interval: 0.625 mm. Images were transmitted to the GE ADW4.7 workstation for further processing and analysis.

#### 2.2.2 CT image processing and analysis

##### 2.2.2.1 ROI image segmentation

CT images were imported into ITK-SNAP software^1^ for image segmentation. Two radiologists with over 5 years of experience, both qualified for pneumoconiosis diagnosis, manually delineated the ROIs for each case. For large shadow lesions, the long diameter was ≥ 20 mm and the short diameter > 10 mm; for peripheral lung cancer lesions, the size was ≥ 3 cm. Each slice was carefully outlined to capture the entire lesion. Completed ROIs were saved in the nii.gz format in the “images” and “mask” directories. A total of 110 ROIs were outlined from the CT images of 103 pneumoconiosis patients, and 85 ROIs were outlined from 85 peripheral lung cancer patients.

To assess the reproducibility of ROI delineation, the inter-observer consistency of the radiomics features was evaluated using the Intraclass Correlation Coefficient (ICC). An ICC value > 0.75 indicated good consistency between the two radiologists (20).

##### 2.2.2.2 Feature selection

Radiomic features were extracted using the Pyradiomics software.^2^ The following feature extraction methods were applied: ➀ Neighborhood Gray Tone Difference Matrix (NGTDM); ➁ Shape features; ➂ First-order features; ➃ Gray-Level Co-occurrence Matrix (GLCM); ➄ Gray-Level Dependence Matrix (GLDM); ➅ Gray-Level Run-Length Matrix (GLRLM); ➆ Gray-Level Size-Zone Matrix (GLSZM). Features for large shadows and peripheral lung cancer were labeled as 0 and 1, respectively.

##### 2.2.2.3 Data processing

To minimize the impact of feature dimensionality and improve model performance, the data were standardized using Z-scores for normalization.

##### 2.2.2.4 Feature screening

For normally distributed features, a t-test was applied, while for non-normally distributed features, a U-test was used (*p* < 0.05 was considered statistically significant). Following screening, 85 features were retained for further analysis.

##### 2.2.2.5 Feature dimensionality reduction

To reduce the dimensionality of the initially extracted 85 radiomic features, Pearson’s correlation analysis was first performed to assess inter-feature relationships. Features exhibiting high pairwise correlation (|r| > 0.9) were removed to minimize multicollinearity and redundancy. Subsequently, Least Absolute Shrinkage and Selection Operator (LASSO) regression was employed to perform penalized feature selection and identify the most informative predictors. The optimal regularization parameter (λ) was determined using 10-fold cross-validation, where the value minimizing the mean binomial deviance was selected to balance model simplicity and predictive performance. The resulting optimal λ value (λ = 0.0222) achieved the lowest deviance and ensured a parsimonious and stable model (Table 1).

**TABLE 1:** Selected radiomic features and corresponding regression coefficients after dimensionality reduction using the LASSO regression model.

Type	Feature name
Selected gray-level non-uniformity texture features (Ngtdm)	original_ngtdm_Busyness, original_ngtdm_Complexity, original_ngtdm_Contrast
Shape features (shape)	original_shape_Maximum3DDiameter, original_shape_Sphericity
Gray-level co-occurrence matrix (Glcm)	original_glcm_ClusterShade, original_glcm_MCC
Gray-level size-zone matrix (Glszm)	original_glszm_SmallAreaLowGrayLevelEmphasis

**FIGURE 3:**
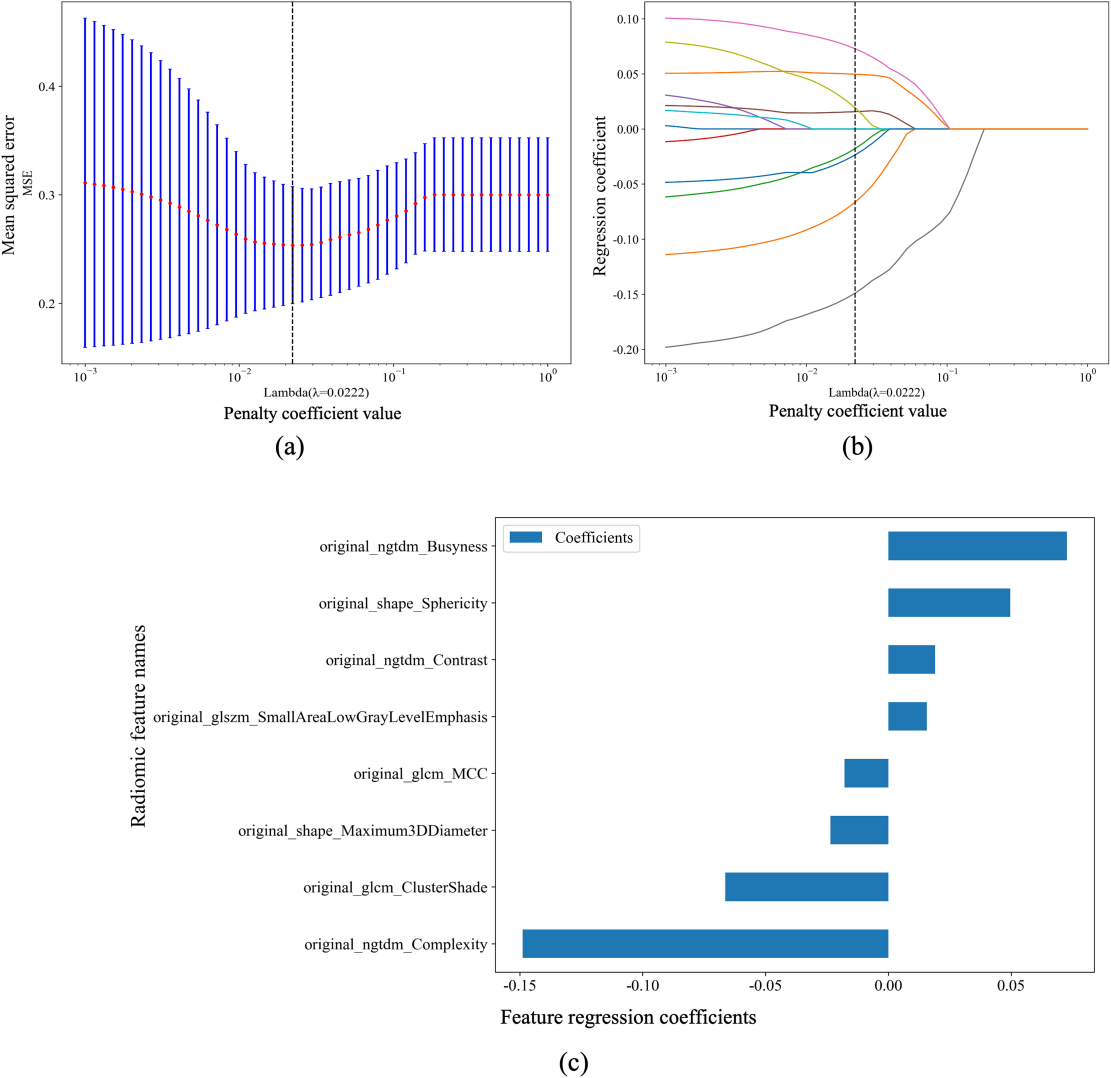
Penalized feature selection and regression coefficients for pneumoconiosis large opacities and peripheral lung cancer classification. **(a)** The relationship between the penalty coefficient value (λ) and the binomial deviance (Y-axis), with the X-axis showing the corresponding number of features. The vertical dashed line indicates the optimal λ value (λ = 0.0222). **(b)** The regression coefficients for each feature plotted against the penalty coefficient value (λ), illustrating the shrinkage effect of the penalty term and the evolution of feature coefficients with increasing λ. **(c)** A horizontal bar chart displaying the selected features with their corresponding regression coefficients. The Y-axis shows the radiomic feature names, and the X-axis represents the magnitude and direction of the coefficients, where bars to the right indicate positive coefficients and those to the left indicate negative coefficients.

As shown in Figure 3a, the X-axis represents the penalty coefficient (λ) and the Y-axis denotes the corresponding mean binomial deviance. The vertical dashed line marks the optimal λ value (λ = 0.0222), indicating the point of minimal deviance. Figure 3b displays the trajectories of regression coefficients for all features as λ increases, illustrating how stronger penalization gradually shrinks less relevant coefficients toward zero. Figure 3c summarizes the final eight selected radiomic features and their standardized regression coefficients, where positive coefficients (bars extending to the right) and negative coefficients (bars extending to the left) indicate their respective contributions to the predictive model.

### 2.3 Statistical processing

Data analysis was performed using the onekey V5.1.25 platform for imaging feature extraction. The Kolmogorov-Smirnov test was used to assess the normality of measurement data. Data following a normal distribution were expressed as means ± standard deviation (x ± s), while non-normally distributed data were reported as medians. The independent *t*-test or Mann-Whitney U test was used for group comparisons, as appropriate. Categorical data were expressed as percentages, and the chi-square test (χ^2^ test) was employed for inter-group comparisons. A *P* < 0.05 was considered statistically significant. The MedCalc version 20.101 software was used to evaluate the efficacy of the diagnostic model by calculating the area under the Receiver Operating Characteristic (ROC) curve (AUC).

## 3 Results

### 3.1 General patient data

A total of 188 patients were enrolled in this study, consisting of 103 patients in the pneumoconiosis large shadow group (all male, with a mean age of 58.37 ± 7.34 years) and 85 patients with peripheral lung cancer (male-to-female ratio approximately 2.6:1, with a mean age of 70.95 ± 10.82 years). Among the 103 pneumoconiosis patients with large shadows, the pathological diagnoses included 32 cases of lung adenocarcinoma, 15 cases of squamous cell carcinoma, 1 case of neuroendocrine carcinoma, 1 case of adenosquamous carcinoma, 6 cases of small cell lung cancer, 2 cases of non-small cell lung cancer, and 28 cases where the pathological type remained unknown. These patients had all been diagnosed with stage III pneumoconiosis, and the large shadows were confirmed as massive fibrosis by expert clinical evaluation and imaging analysis. For the peripheral lung cancer group, all cases were pathologically confirmed after biopsy and surgery. The study population exhibited a range of underlying conditions, with some patients presenting with comorbidities such as chronic obstructive pulmonary disease (COPD) or cardiovascular disease, which were considered during the analysis to prevent bias in the feature extraction process.

### 3.2 Construction of classifiers

The 188-patient dataset was randomly divided into a training set and a validation set at a ratio of 7:3, resulting in 132 patients for the training set and 56 for the validation set. Several common machine learning algorithms were employed to construct predictive models, using the onekey AI V5.1.25 platform: (1) Logistic Regression (LR): a generalized linear model that maps the linear regression output to a probability space through a logistic function, which is used for classification purposes. LR is widely used for its interpretability and simplicity, making it a strong baseline method for binary classification tasks. (2) Support Vector Machine (SVM): a supervised learning algorithm that performs classification by finding the optimal hyperplane that maximizes the margin between different classes. SVM is effective for classifying complex, non-linear data and is particularly useful for high-dimensional feature spaces, such as the radiomic features extracted from CT images. (3) Adaptive Boosting (AdaBoost): an ensemble learning method that combines multiple weak classifiers to form a strong classifier. By giving higher weights to misclassified samples in each iteration, AdaBoost improves the overall model accuracy. Notably, AdaBoost is known for its ability to reduce overfitting and enhance the generalization capability of the model, even when training errors approach zero.

### 3.3 Model performance results

The performance of each model was evaluated based on various metrics, including accuracy, AUC (Area Under the Curve), sensitivity, specificity, precision, recall, F1 score, and predictive values. The detailed results for each model are as follows:

LR Model: ➀ accuracy: 79.4%; ➁ AUC: 0.837 (95% CI: 0.7687-0.9059); ➂ sensitivity: 74.1%; ➃ specificity: 83.6%; ➄ positive Predictive Value (PPV): 78.2%; ➅ precision: 78.2%; ➆ Negative Predictive Value (NPV): 80.3%; Recall: 74.1%; ➇ F1 Score: 76.1%.

SVM Model: ➀ accuracy: 84.0%; ➁ AUC: 0.886 (95% CI: 0.8277–0.9437); ➂ sensitivity: 74.1%; ➃ specificity: 91.8%; ➄ PPV: 87.8%; ➅ precision: 87.8%; ➆ NPV: 81.7%; ➇ recall: 74.1%; ➈ F1 Score: 80.4%.

AdaBoost Model: ➀ accuracy: 80.9%; ➁ AUC: 0.900 (95% CI: 0.8498–0.9497); ➂ sensitivity: 81.0%; ➃ specificity: 80.8%; ➄ PPV: 77.0%; ➅ precision: 77.0%; ➆ NPV: 84.3%; ➇ recall: 81.0%; ➈ F1 Score: 79.0% (Table 2).

**TABLE 2:** Performance metrics of LR, SVM, and AdaBoost models in the training and test sets.

Model_name	Accuracy	AUC	95% CI	Sensitivity	Specificity	PPV	NPV	F1
LR (train)	0.794	0.837	0.7687–0.9059	0.741	0.836	0.782	0.803	0.761
LR (test)	0.807	0.825	0.7121–0.9381	0.893	0.724	0.758	0.875	0.82
SVM (train)	0.84	0.886	0.8277–0.9437	0.741	0.918	0.878	0.817	0.804
SVM (test)	0.825	0.855	0.7547–0.9547	0.893	0.759	0.781	0.88	0.833
AdaBoost (train)	0.809	0.900	0.8498–0.9497	0.810	0.808	0.770	0.843	0.790
AdaBoost (test)	0.860	0.869	0.7634–0.9755	0.821	0.897	0.885	0.839	0.852

The AdaBoost model achieved the highest performance across all metrics, with an accuracy of 80.9% and an AUC of 0.900, suggesting its superior ability to distinguish between pneumoconiosis large opacities and peripheral lung cancer. The SVM model also demonstrated robust performance with an accuracy of 84.0% and an AUC of 0.886, while the LR model provided a solid baseline, achieving an accuracy of 79.4% and an AUC of 0.837.

### 3.4 Net benefit and clinical relevance

Further evaluation of the models’ clinical relevance was performed by calculating the net benefit within different probability threshold ranges. In the low threshold probability range (0–0.4), the AdaBoost model showed a significantly higher net benefit compared to the “treat all” strategy, indicating its capacity to accurately identify patients who would benefit from early intervention. In the moderate threshold probability range (0.4–0.6), the net benefit of the AdaBoost model remained higher than both the “treat all” and “treat none” strategies, suggesting its substantial clinical predictive value.

**FIGURE 4:**
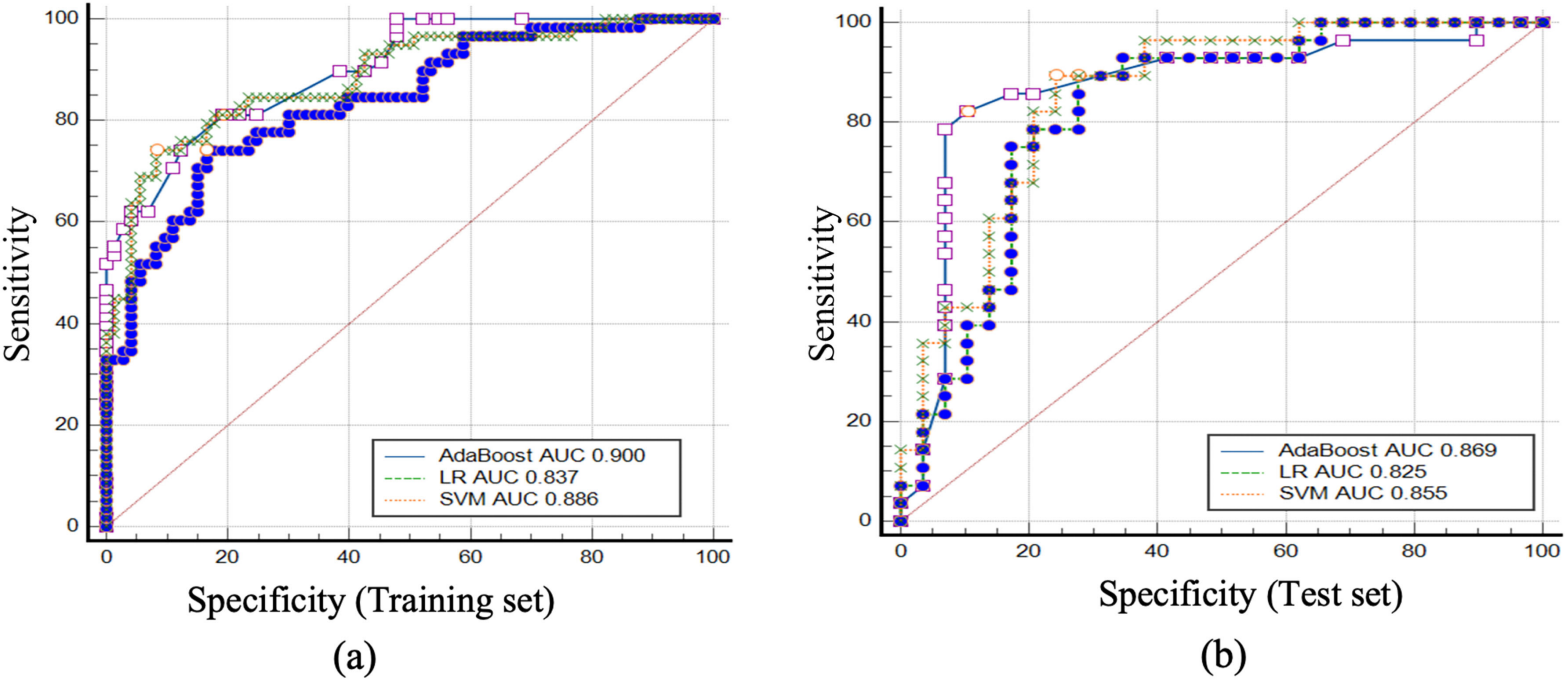
ROC curves and AUC values for the AdaBoost, LR, and SVM models in predicting pneumoconiosis large opacities and peripheral lung cancer in the training and test sets. **(a)** the ROC curves and corresponding AUC values for the AdaBoost, LR, and SVM models in the training set, with the AUC values for AdaBoost, LR, and SVM being 0.900, 0.837, and 0.886, respectively. **(b)** the ROC curves and AUC values for the models in the test set, with the AUC values for AdaBoost, LR, and SVM being 0.869, 0.825, and 0.855, respectively. These results demonstrate the performance of each model in differentiating between pneumoconiosis large opacities and peripheral lung cancer, highlighting AdaBoost as the top performer in both the training and test sets.

**FIGURE 5:**
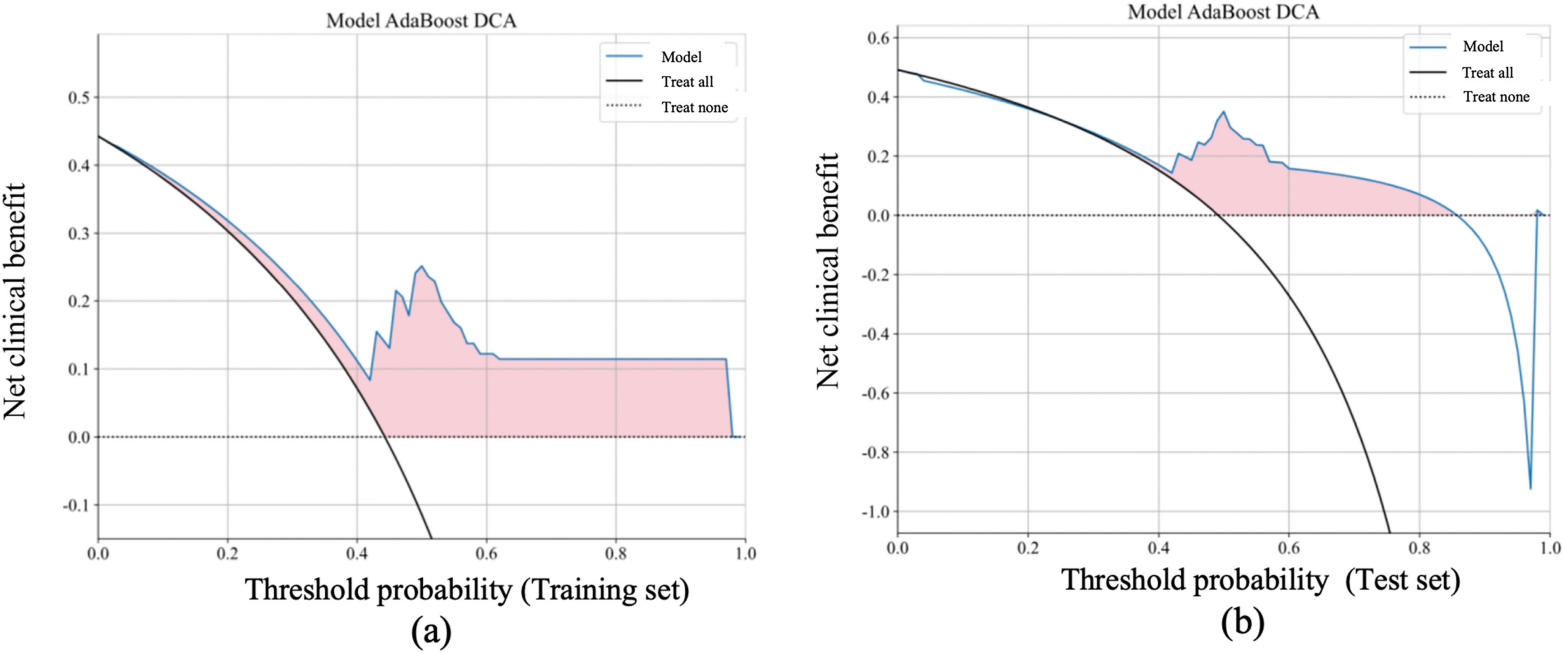
Net benefit of the AdaBoost model in the training and test sets based on DCA Curves, compared with “Treat all” and “Treat none” strategies. The DCA curves for the AdaBoost model, showing the net benefit in both the training **(a)** and test sets **(b)** at various threshold probabilities. The net benefit is calculated as the correct decision gain minus the loss from incorrect decisions. The horizontal axis represents the threshold probability, which indicates the minimum probability threshold for classifying a sample as positive. The vertical axis represents the net benefit. The model’s performance (blue line) is compared with the “treat all” (black line) and “treat none” (dashed black line) strategies. The shaded pink area indicates the range where the AdaBoost model provides a higher net benefit compared to both “treat all” and “treat none” strategies. The AdaBoost model demonstrates significantly higher net benefit in the low threshold probability range (0–0.4), indicating its ability to effectively identify patients who require treatment at an early stage. In the moderate threshold range (0.4–0.6), the model still outperforms both the “treat all” and “treat none” strategies, suggesting its predictive value for clinical decision-making.

Figures 4, 5 illustrate the detailed net benefit curves for each model across different probability thresholds, providing a comprehensive view of their decision-making potential. These figures demonstrate the AdaBoost model’s superior performance in maximizing clinical benefit while minimizing unnecessary treatments.

## 4 Discussion

Radiomics enables the quantitative extraction and analysis of high-dimensional features from medical imaging data, providing a non-invasive, reproducible, and multidimensional assessment of disease characteristics (21). By integrating multimodal and multiparametric information, radiomics facilitates early diagnosis, individualized treatment planning, and disease monitoring, thus serving as a cornerstone of precision medicine (22).

In the context of pneumoconiosis and lung cancer, the diagnostic challenge arises from the striking similarity in their radiologic manifestations, particularly in Stage III occupational pneumoconiosis with progressive massive fibrosis (PMF) (23). Advanced fibrotic lesions in PMF often exhibit imaging characteristics, e.g., abnormal CT attenuation, calcification, satellite nodules, spiculation, pleural thickening, and cavitation-that closely mimic those of peripheral lung malignancies (24). The situation becomes even more complex when pneumoconiosis coexists with lung cancer, which may lead to misinterpretation and delayed diagnosis (25). Epidemiological data suggest that approximately 3.2% of patients with simple pneumoconiosis progress to PMF over an 8-year follow-up, and these patients have higher mortality rates than those with uncomplicated pneumoconiosis (26). Therefore, accurate differentiation between PMF-related large opacities and lung cancer is crucial for guiding clinical management and preventing inappropriate or delayed interventions (27).

Previous studies have explored the use of supplementary imaging modalities, such as MRI, including T2-weighted and diffusion-weighted imaging, to distinguish PMF from malignancies t (28). However, MRI is often impractical in patients with advanced pneumoconiosis, who typically exhibit severe respiratory impairment (29). This limitation underscores the urgent need for non-invasive, efficient, and clinically applicable diagnostic tools (30). In this regard, artificial intelligence (AI)-based radiomics and machine learning approaches have shown significant promise in decoding complex imaging patterns that exceed human perceptual capability (31). Prior investigations, such as those by Warkentin et al. and Dong et al., demonstrated the potential of ML algorithms in predicting lung nodule malignancy and assessing pneumoconiosis risk, respectively (32, 33). Collectively, these findings establish a foundation for applying AI-driven radiomics to differential diagnosis in occupational lung diseases.

The present study developed and validated multiple ML-based diagnostic models-including AdaBoost, LR, and SVM-to differentiate pneumoconiosis-associated large opacities from peripheral lung cancer. Among these, the AdaBoost model demonstrated superior performance, achieving an accuracy of 80.9%, sensitivity of 81.0%, specificity of 80.8%, and an area under the ROC curve (AUC) of 0.900. These results affirm the feasibility of integrating radiomic features with ML algorithms to construct clinically valuable, non-invasive predictive models. Importantly, DCA further verified the net clinical benefit of the proposed models, surpassing both “treat-all” and “treat-none” strategies across a broad range of threshold probabilities (34).

From a methodological standpoint, our study highlights the synergistic potential of combining traditional statistical models (e.g., LR) with more advanced ensemble learning techniques (e.g., AdaBoost and SVM) (35). This hybrid approach leverages both interpretability and predictive power, offering a balanced framework for clinical implementation. The inclusion of LASSO regression and Pearson correlation for dimensionality reduction ensured that only the most relevant, non-redundant features were retained, optimizing model performance and generalizability (36).

Nevertheless, several limitations warrant acknowledgment. Firstly, this was a single-center, retrospective study, which may introduce selection bias and limit external generalizability (37). Secondly, although the models achieved satisfactory accuracy, external validation using independent, multicenter datasets is essential to confirm robustness and clinical applicability (38). Thirdly, the current analysis relied solely on imaging-derived features and basic clinical parameters, without incorporating molecular, genomic, or proteomic biomarkers (39). Integrating multi-omics data in future studies could further enhance diagnostic precision and biological interpretability (39). Additionally, the implementation of explainable AI (XAI) frameworks may help elucidate model decision pathways, thereby increasing clinician confidence and promoting real-world adoption (40).

## 5 Conclusion

In conclusion, this study demonstrates that machine learning–based radiomic models, particularly the AdaBoost algorithm, can effectively differentiate between pneumoconiosis with large opacities and peripheral lung cancer, achieving robust diagnostic accuracy and measurable clinical benefit. With further multicenter validation and model interpretability enhancements, such AI-driven approaches hold great potential as practical, non-invasive decision-support tools in occupational and oncologic respiratory medicine, ultimately contributing to more accurate diagnosis, optimized treatment strategies, and improved patient outcomes (41).

## Data Availability

The original contributions presented in this study are included in this article/Supplementary material, further inquiries can be directed to the corresponding authors.

## Footnotes

1
http://www.itksnap.org/

2
http://pyradiomics.readthedocs.io

## Supplementary material

Supplementary Figure 1 Representative CT images showing different stages of pneumoconiosis (axial and coronal views). **(a,b)** Axial and coronal chest CT images of a Stage I pneumoconiosis patient demonstrate multiple scattered, small, round, high-density nodules distributed in both lungs, predominantly in the right middle lung field. **(c,d)** Stage II pneumoconiosis is characterized by an increased number of small nodules and linear opacities, primarily clustered in the posterior segment of the right upper lobe. **(e,f)** Stage III pneumoconiosis exhibits more extensive and symmetrical large opacities (long diameter > 20 mm, short diameter > 10 mm) in the apical and posterior segments of both upper lobes. The lesions are accompanied by pleural thickening, traction, and partial calcification within the masses, along with multiple peripheral nodular opacities. *Interpretation*: as pneumoconiosis progresses from Stage I to Stage III, CT imaging reveals a gradual increase in nodule size and density, evolving into confluent fibrotic masses with pleural involvement and architectural distortion, consistent with advanced PMF.

Supplementary Figure 2 CT imaging and immunohistochemical (IHC) profiles of different histological types of lung cancer. **(a,b)** Axial and coronal CT images of squamous cell carcinoma in the left upper lobe reveal a soft-tissue mass located in the anterior segment, exhibiting short spiculation and well-defined margins. IHC profile: CK (+), CK7(+), CK5/6(few +), TTF-1(−), NapsinA(−), CgA(−), Syn(−), P40(+), P63(+), CDX-2(−), CK20(−), Ki-67 (∼80% +), Villin(−). Diagnosis: Squamous cell carcinoma of the lung. **(c)** Axial CT image of poorly differentiated adenocarcinoma in the right middle lobe shows an ill-defined mass with partial truncation of the middle bronchus. IHC profile: CK (+), CK7(+), TTF-1(+), NapsinA(−), CgA(−), Syn(−), CD56(−), INSM-1(+), CK5/6(−), P40(−), P63(−), Ki-67 (∼70% +). Diagnosis: Poorly differentiated adenocarcinoma. **(d)** Axial CT image of a poorly differentiated small cell carcinoma in the right lower lobe demonstrates a large, irregular mass with unclear margins, bronchial obstruction, and significant mass effect. IHC profile: CK (+), Ki-67 (∼60% +), CK7(+), P63(−), P40(−), TTF-1(−), NapsinA(−), Syn(weak +), CgA(−), CK5/6(weak +), INSM-1(−), CD56(weak +), HMB-45(−), LCA(−), INI-1(+), S100(−), NUT(−), CD20(−). Diagnosis: Poorly differentiated small cell carcinoma confirmed by immunohistochemistry. *Interpretation*: Distinct CT and immunohistochemical patterns are evident among different lung cancer subtypes. While squamous cell carcinoma typically presents as a well-circumscribed lesion with peripheral spiculation, adenocarcinoma and small cell carcinoma often exhibit irregular borders, bronchial involvement, and aggressive radiologic behavior. Combined radiologic and IHC assessment remains essential for accurate histopathological classification.
