# Supplementary figures and images for: Differential diagnosis of pneumoconiosis mass shadows and peripheral lung cancer using CT radiomics and the AdaBoost machine learning model
Source: Front Med (Lausanne). 2025 Dec 3;12:1675840. doi: 10.3389/fmed.2025.1675840 (PMC12740114; doi:10.3389/fmed.2025.1675840)

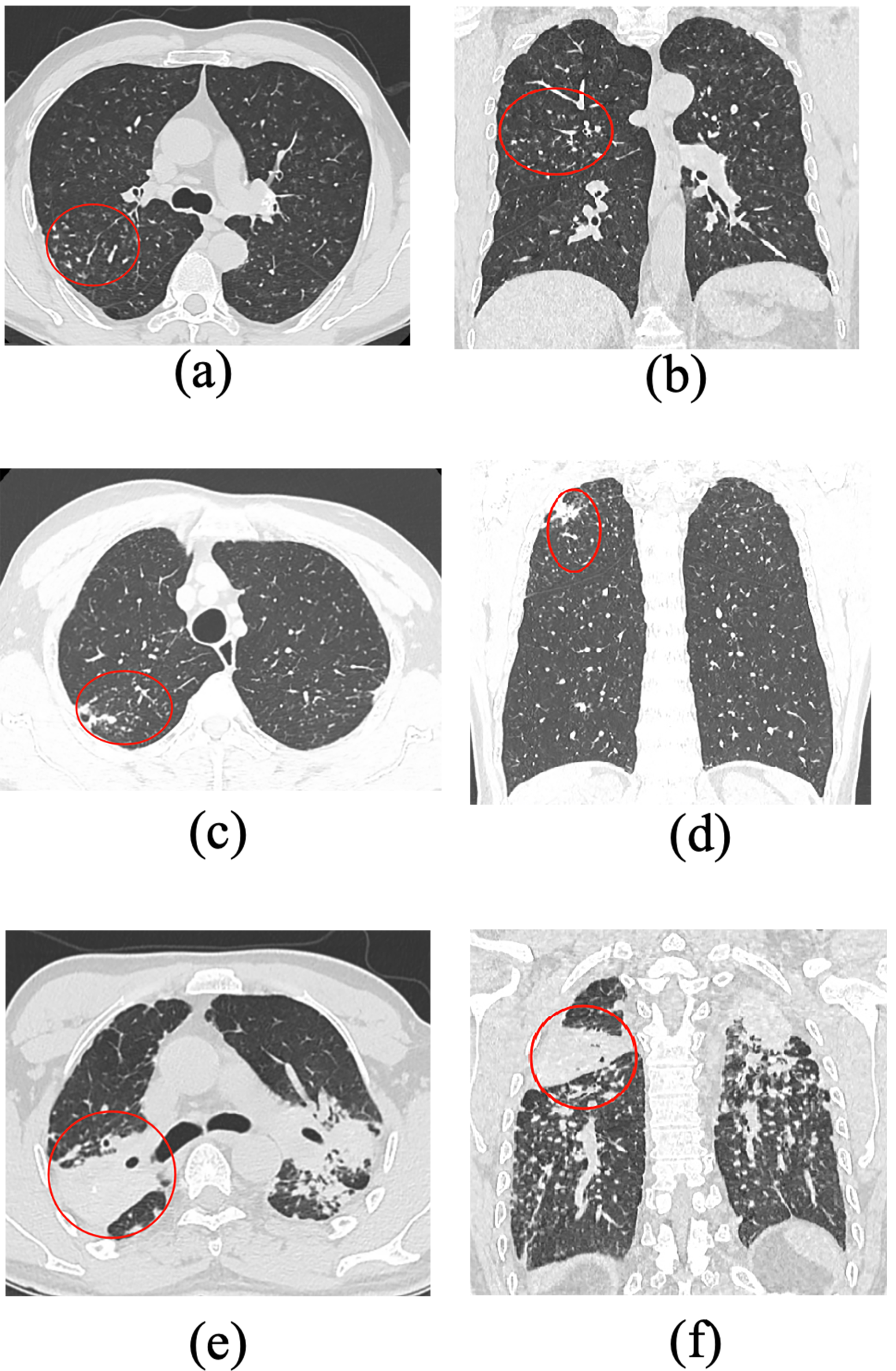

Supplement: Supplementary Figure 1 — Representative CT images showing different stages of pneumoconiosis (axial and coronal views). (a,b) Axial and coronal chest CT images of a Stage I pneumoconiosis patient demonstrate multiple scattered, small, round, high-density nodules distributed in both lungs, predominantly in the right middle lung field. (c,d) Stage II pneumoconiosis is characterized by an increased number of small nodules and linear opacities, primarily clustered in the posterior segment of the right upper lobe. (e,f) Stage III pneumoconiosis exhibits more extensive and symmetrical large opacities (long diameter > 20 mm, short diameter > 10 mm) in the apical and posterior segments of both upper lobes. The lesions are accompanied by pleural thickening, traction, and partial calcification within the masses, along with multiple peripheral nodular opacities. Interpretation: as pneumoconiosis progresses from Stage I to Stage III, CT imaging reveals a gradual increase in nodule size and density, evolving into confluent fibrotic masses with pleural involvement and architectural distortion, consistent with advanced PMF. [file Image_1.tif]

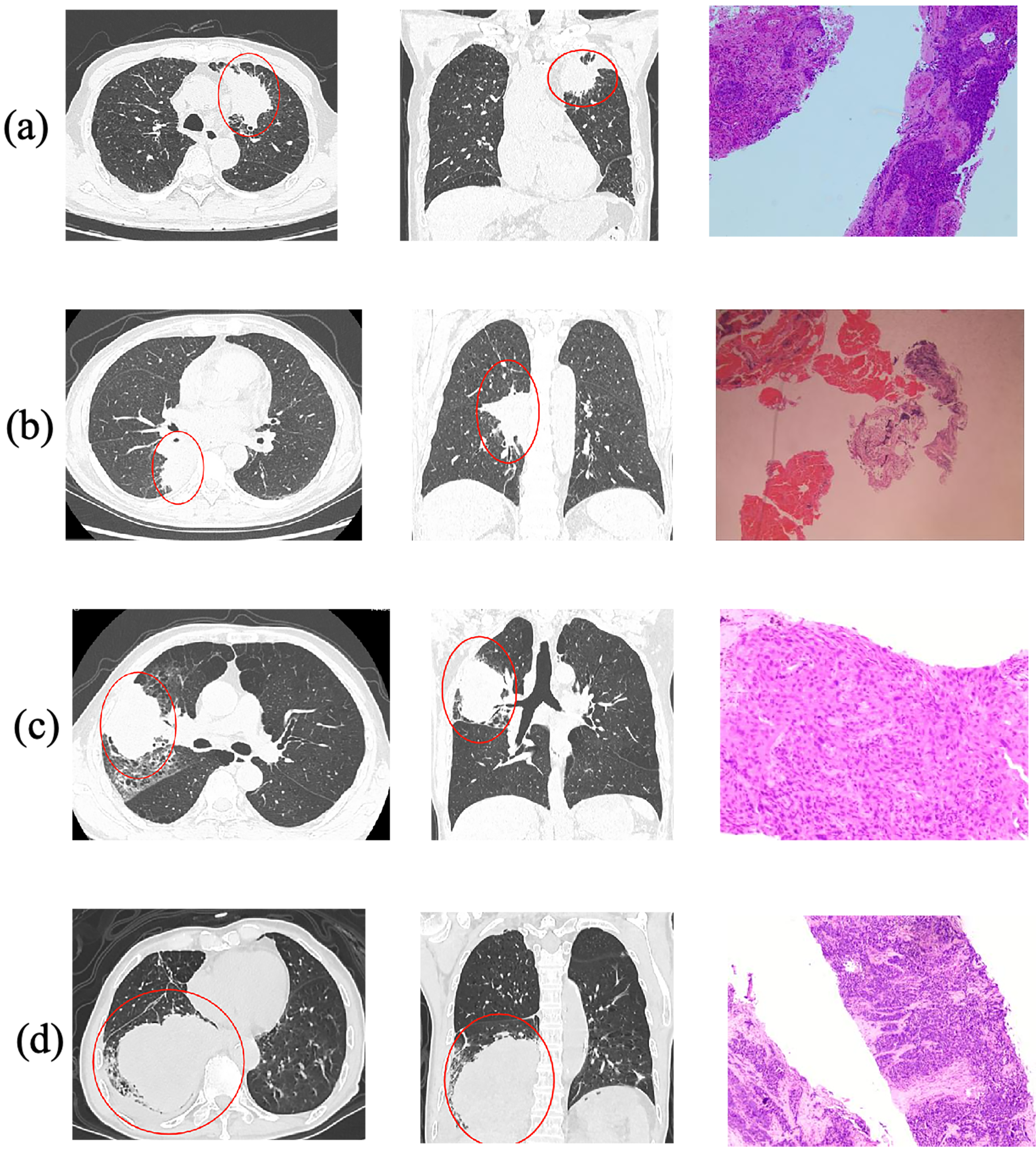

Supplement: Supplementary Figure 2 — CT imaging and immunohistochemical (IHC) profiles of different histological types of lung cancer. (a,b) Axial and coronal CT images of squamous cell carcinoma in the left upper lobe reveal a soft-tissue mass located in the anterior segment, exhibiting short spiculation and well-defined margins. IHC profile: CK (+), CK7(+), CK5/6(few +), TTF-1(−), NapsinA(−), CgA(−), Syn(−), P40(+), P63(+), CDX-2(−), CK20(−), Ki-67 (∼80% +), Villin(−). Diagnosis: Squamous cell carcinoma of the lung. (c) Axial CT image of poorly differentiated adenocarcinoma in the right middle lobe shows an ill-defined mass with partial truncation of the middle bronchus. IHC profile: CK (+), CK7(+), TTF-1(+), NapsinA(−), CgA(−), Syn(−), CD56(−), INSM-1(+), CK5/6(−), P40(−), P63(−), Ki-67 (∼70% +). Diagnosis: Poorly differentiated adenocarcinoma. (d) Axial CT image of a poorly differentiated small cell carcinoma in the right lower lobe demonstrates a large, irregular mass with unclear margins, bronchial obstruction, and significant mass effect. IHC profile: CK (+), Ki-67 (∼60% +), CK7(+), P63(−), P40(−), TTF-1(−), NapsinA(−), Syn(weak +), CgA(−), CK5/6(weak +), INSM-1(−), CD56(weak +), HMB-45(−), LCA(−), INI-1(+), S100(−), NUT(−), CD20(−). Diagnosis: Poorly differentiated small cell carcinoma confirmed by immunohistochemistry. Interpretation: Distinct CT and immunohistochemical patterns are evident among different lung cancer subtypes. While squamous cell carcinoma typically presents as a well-circumscribed lesion with peripheral spiculation, adenocarcinoma and small cell carcinoma often exhibit irregular borders, bronchial involvement, and aggressive radiologic behavior. Combined radiologic and IHC assessment remains essential for accurate histopathological classification. [file Image_2.tif]
